# The intestinal microbiota determines the colitis‐inducing potential of T‐bet‐deficient Th cells in mice

**DOI:** 10.1002/eji.201747100

**Published:** 2017-09-29

**Authors:** Jakob Zimmermann, Pawel Durek, Anja A. Kühl, Florian Schattenberg, Patrick Maschmeyer, Francesco Siracusa, Katrin Lehmann, Kerstin Westendorf, Melanie Weber, René Riedel, Susann Müller, Andreas Radbruch, Hyun‐Dong Chang

**Affiliations:** ^1^ Deutsches Rheumaforschungszentrum Berlin (DRFZ) an Institute of the Leibniz Association Berlin Germany; ^2^ Charité Universitätsmedizin Campus Benjamin Franklin Berlin Germany; ^3^ Helmholtzzentrum für Umweltforschung (UFZ) Leipzig Department Umweltmikrobiologie Leipzig Germany; ^4^ Maurice Müller Laboratories (DKF) Universitätsklinik für Viszerale Chirurgie & Medizin Inselspital University of Bern Bern Switzerland; ^5^ Max Planck Institute for Evolutionary Biology Plön Germany

**Keywords:** Inflammatory bowel disease, Microbiota, T cell transfer colitis, T helper cells, T‐bet

## Abstract

Conflicting evidence has been provided as to whether induction of intestinal inflammation by adoptive transfer of naïve T cells into *Rag* ^−/−^ mice requires expression of the transcription factor T‑bet by the T cells. Here, we formally show that the intestinal microbiota composition of the *Rag* ^−/−^ recipient determines whether or not T‐bet‐deficient Th cells can induce colitis and we have resolved the differences of the two microbiomes, permissive or non‐permissive to T‐bet‐independent colitis. Our data highlight the dominance of the microbiota over particular T cell differentiation programs in the pathogenesis of chronic intestinal inflammation.

## Introduction

Crohn's disease – a chronic inflammatory disorder of the gastrointestinal tract – is characterized by the accumulation of proinflammatory T helper (Th) 1, Th17, and bifunctional Th1/17 cells in the intestinal mucosa 1. However, the relative contribution of the various T cell subsets to disease initiation and perpetuation has remained elusive. In mice, intestinal inflammation can be induced by transfer of naïve CD45RB^hi^ Th cells into immunodeficient *Rag* ^−/−^ mice 2. Similarly to Crohn's disease, transferred T cells differentiate into Th1, Th17, and hybrid Th1/17 cells and cause inflammation by recruiting and activating myeloid effector cells 3, 4, 5. However, conflicting evidence has been provided as to whether induction of colitis is dependent on expression of the Th1 lineage‐determining transcription factor T‐bet by the Th cells 5, 6, 7, 8, 9. Here we show that the recipient's intestinal microbiota determines whether T‐bet‐deficient Th cells can induce colitis.

## Results and discussion

### Recipient hygiene status determines colitis‐inducing potential of T‐bet‐deficient Th cells

**Figure 1:**
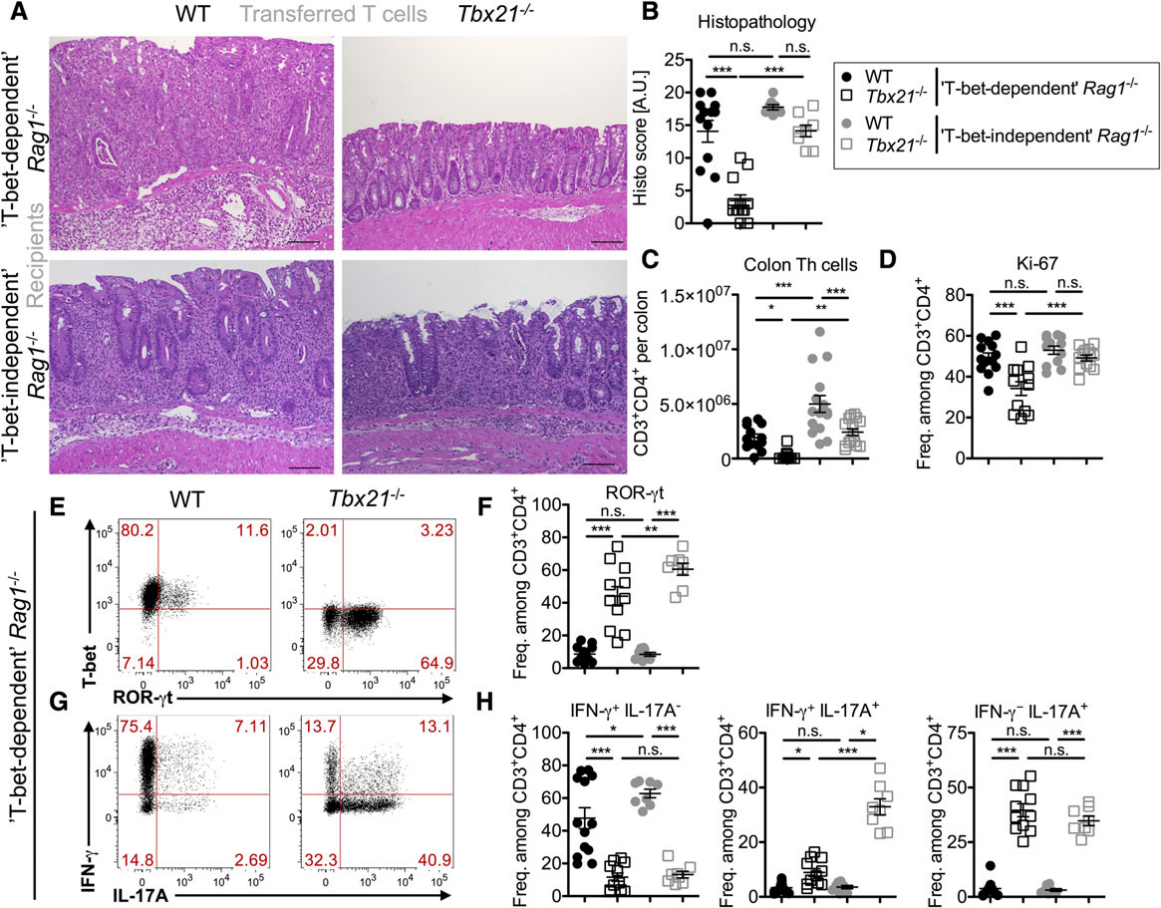
The microbiota determines the colitis‐inducing potential of T‐bet‐deficient Th cells. CD4^+^CD45RB^hi^CD25^−^ WT or *Tbx21* ^−/−^ CD4 T cells were transferred into *Rag1^−/−^* recipients directly from Charles River (‘T‐bet‐dependent’ *Rag1* ^−/−^) or after colonization with fecal microbiota from the DRFZ animal colony (‘T‐bet‐independent’ *Rag1* ^−/−^). (A) Representative hematoxylin‐eosin staining of the colon on day 40–44 (‘T‐bet‐dependent’ *Rag1* ^−/−^) and day 18 (‘T‐bet‐independent’ *Rag1* ^−/−^), bars are 100 μm. (B) Colon histopathology scores for *n* = 13 ‘T‐bet‐dependent’ recipients per group pooled from three experiments and *n* = 8 ‘T‐bet‐independent’ *Rag1* ^−/−^ recipients per group pooled from two experiments. (C) Absolute number of colonic CD4^+^ Th cells for *n* = 12‐13 ‘T‐bet‐dependent’ recipients per group pooled from three experiments and n = 15 ‘T‐bet‐independent’ *Rag1* ^−/−^ recipients per group pooled from four experiments (full gating strategy in Supporting Information Fig. 1). (D) Frequency of Ki‐67‐expressing colonic CD4^+^ Th cells for *n* = 12‐13 mice per group each pooled from three experiments. (E, G) Expression of T‐bet/ROR‐γt and IFN‐γ/IL‐17A by colonic CD4^+^ Th cells from ‘T‐bet‐dependent’ *Rag1* ^−/−^ recipients. (F, H) Frequency of ROR‐γt and cytokine‐expressing colonic CD4^+^ Th cells from *n* = 11‐13 ‘T‐bet‐dependent’ recipients pooled from three experiments and *n* = 8 ‘T‐bet‐independent’ *Rag1* ^−/−^ recipients pooled from two experiments. (B‐D, F, H) Data are shown as $\bar{x}$±SEM with ^*^ *p*<0.05, ^**^ *p*<0.01, and ^***^ *p*<0.001 by one‐way ANOVA and Newman–Keuls test. Data for ‘T‐bet‐independent’ *Rag1* ^−/−^ mice are from 5.

When transferring CD4^+^CD45RB^hi^CD25^−^ Th cells into *Rag1* ^−/−^ recipients, derived from the Jackson Laboratory (USA) and bred at a Charles River facility (Sulzfeld, Germany), WT but not T‐bet‐deficient Th cells induced colitis (Fig. 1A, B ‘T‐bet‐dependent’ *Rag1* ^−/−^). When instead *Rag1* ^−/−^ recipients were transplanted with fecal microbiota from the DRFZ Berlin animal facility prior to T cell transfer, T‐bet‐deficient Th cells induced colitis comparable to WT Th cells (Fig. 1A, B ‘T‐bet‐independent’ *Rag1* ^−/−^ from ref. 5). In ‘T‐bet‐dependent’ *Rag1* ^−/−^ recipients, absolute numbers of T‐bet‐deficient Th cells were 8‐times lower compared with WT Th cells (Fig. 1C – black symbols). The frequency of Th cells expressing the cell cycle marker Ki‐67 was significantly higher for WT compared with T‐bet‐deficient Th cells (Fig. 1D – black symbols). These observations contrast the results obtained with ‘T‐bet‐independent’ *Rag1* ^−/−^ recipients in which absolute numbers of T‐bet‐deficient Th cells reached approximately 50% of the WT level and Ki‐67 expression was similar between WT and T‐bet‐deficient Th cells (Fig. 1C, D – gray symbols, from ref. 5).

In ‘T‐bet‐dependent’ *Rag1* ^−/−^ mice, the frequency of T‐bet‐deficient ROR‐γt‐expressing Th cells was strongly increased compared with WT but remained below the frequency of ROR‐γt^+^ T‐bet‐deficient Th cells in ‘T‐bet‐independent’ *Rag1* ^−/−^ recipients (Fig. 1E, F). In both types of recipients, T‐bet‐deficient Th cells had decreased IFN‐γ and increased IL‐17A expression compared with WT Th cells (Fig. 1G, H). ‘T‐bet‐dependent’ *Rag1* ^−/−^ recipients had reduced frequencies of IFN‐γ^+^IL‐17A^+^ double‐positive T‐bet‐deficient Th cells when compared with ‘T‐bet‐independent’ *Rag1* ^−/−^ recipients (Fig. 1H). Relative frequencies of Foxp3^+^ regulatory T cells were increased in the colon of ‘T‐bet‐dependent’ *Rag1* ^−/−^ recipients after transfer of T‐bet‐deficient compared with WT Th cells. However, absolute numbers of Foxp3^+^ regulatory T cells remained similar (Supporting Information Fig. 2).

We have previously described that T‐bet‐deficient Th cells orchestrate a different inflammatory reaction compared with WT with reduced numbers of monocytes and macrophages but increased numbers of eosinophils in the colon of ‘T‐bet‐independent’ *Rag1* ^−/−^ recipients 5. In ‘T‐bet‐dependent’ *Rag1* ^−/−^ recipients, T‐bet‐deficient Th cells induced colitis with strongly reduced numbers of monocytes, macrophages, and neutrophils but similar numbers of eosinophils compared with WT Th cells (Supporting Information Fig. 3).

### Microbiome differences of mice permissive or non‐permissive to T‐bet‐independent colitis

**Figure 2:**
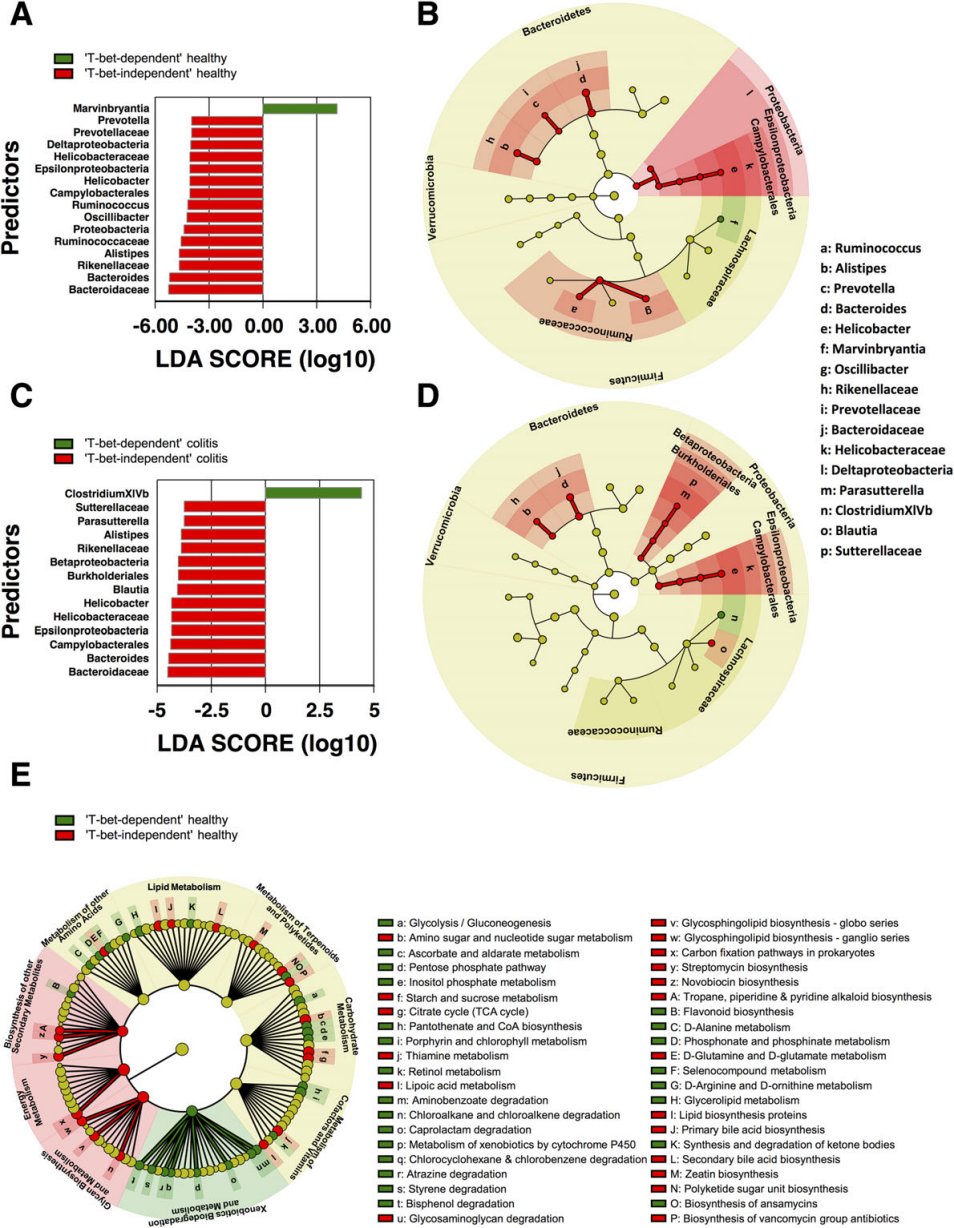
The microbiomes of ‘T‐bet‐dependent’ and ‘T‐bet‐independent’ *Rag1* ^−/−^ recipients. Fecal microbiomes of mice from Fig. 1 were determined by 16S rDNA sequencing for *n* = 5 ‘T‐bet‐dependent’ *Rag1* ^−/−^ mice and *n* = 3 ‘T‐bet‐independent’ *Rag1* ^−/−^ mice from reference 29 before (‘Healthy’) and after (‘Colitis’) transfer of WT Th cells (‘T‐bet‐dependent’ *Rag1* ^−/−^: day 40, ‘T‐bet‐independent’ *Rag1* ^−/−^: day 12–15). (A and C) Linear discriminant analysis (LDA) scores of taxa significantly enriched in ‘T‐bet‐dependent’ (green) or ‘T‐bet‐independent’ (red) microbiomes (A ‐ healthy, C ‐ colitis). Taxa with a relative abundance of at least 1% in at least one sample were included. LDA scores ≥ 2 were considered significant. (B and D) Cladograms showing the phylogenetic relationship among the analyzed taxa at the kingdom, phylum, class, order, and family level with dot size representing the mean abundance of the taxa. (E) PICRUSt analysis depicting KEGG pathways significantly enriched in healthy ‘T‐bet‐dependent’ (green) or ‘T‐bet‐independent’ (red) microbiomes with dot size representing the mean ortholog count of the pathway. LDA scores ≥ 2 were considered significant. Data shown are from each one experiment with *n* = 3 mice (‘T‐bet‐independent’) and *n* = 5 mice (‘T‐bet‐dependent’) per group.

The fecal microbiota composition of *Rag1* ^−/−^ mice enabling ‘T‐bet‐dependent’ *versus* ‘T‐bet‐independent’ colitis was substantially different, both before and after transfer of WT T cells (Fig. 2, Supporting Information Figs. 4–7). Linear discriminant analysis effect size (LEfSe) 10 and random forest 11 analysis identified several genera from three major phyla (*Bacteroidetes*, *Firmicutes*, and *Proteobacteria*) that distinguished ‘T‐bet‐dependent’ from ‘T‐bet‐independent’ microbiomes. Whereas ‘T‐bet‐dependent’ *Rag* ^−/−^ recipients were specifically enriched in *Marvinbryantia* and *Clostridium cluster XIVb*, ‘T‐bet‐independent’ *Rag* ^−/−^ mice revealed an increase in many different taxa including those previously implicated in intestinal inflammation such as *Prevotella* and *Bacteroides* 12, 13 (Fig. 2A and C). Notably, colitis resulted in reduced alpha diversity selectively in ‘T‐bet‐independent’ *Rag1* ^−/−^ recipients (Supporting Information Fig. 4). Among the taxa enriched in ‘T‐bet‐independent’ *Rag* ^−/−^ recipients, we identified *Helicobacter species* including *Helicobacter hepaticus* (Fig. 2A–D, Supporting Information Figs. 5–7) that have previously been shown to drive colitis 14. However, T‐bet‐deficient Th cells induced colitis comparable with WT in *Rag1* ^−/−^ recipients tested negative for *Helicobacter species* by PCR (Supporting Information Fig. 8). In *Helicobacter*‐negative recipients, colitis development was delayed to 38–40 days compared with 18 days in *Helicobacter*‐positive mice (data not shown). Segmented filamentous bacteria (SFB), which have also been implicated in colitis induction 15, were present in ‘T‐bet‐independent’ but not in ‘T‐bet‐dependent’ *Rag1* ^−/−^ recipients as detected by conventional PCR from feces (data not shown). Their presence correlated with increased colonic frequencies of ROR‐γt+ T cells in ‘T‐bet‐independent’ *Rag1* ^−/−^ recipients (Fig. 1F). However, whether SFB determine susceptibility to colitis induced by T‐bet‐deficient Th cells remains elusive and seems unlikely given our previous observations of a protective function of IL‐17 in colitis induced by both WT and T‐bet‐deficient Th cells 5.

Based on the significant differences in microbiota composition between ‘T‐bet‐dependent’ and ‘T‐bet‐independent’ *Rag1* ^−/−^ recipients, we analyzed possible functional differences of the two microbiomes using PICRUSt (phylogenetic investigation of communities by reconstruction of unobserved states) 16. The two microbiomes were predicted to differ strongly in the representation of metabolic pathways (Fig. 2E). Thus, differential susceptibility to colitis induced by T‐bet‐deficient Th cells could not only be mediated by direct bacteria ‐ host interactions 15, 17, 18, but also by bacterial metabolites 19, 20, 21. Overall, it remains to be determined which bacterial taxa differentially activate T‐bet‐sufficient or T‐bet‐deficient Th cells to induce colitis and how they do it, e.g. by microbiota‐derived metabolites or direct interaction with the host.

Collectively, our data show that the requirement for T‐bet for Th cell colitogenicity depends on the composition of the intestinal microbiota of the *Rag1* ^−/−^ recipients. In non‐permissive *Rag1* ^−/−^ recipients T‐bet‐deficient Th cells had reduced proliferative activity and diminished differentiation towards IFN‐γ^+^IL‐17A^+^ Th cells. It has been shown previously that microbiota‐induced T cell proliferation is required for colitis development in this model 22. T‐bet expression seems to overcome the reduced T cell stimulation by certain bacteria which supports a previously described role for T‐bet in enhancing T cell proliferation 23.

Moreover, the permissive microbiota apparently promotes differentiation of T‐bet‐deficient Th cells into IFN‐γ^+^IL‐17A^+^ Th cells, offering another possible mechanism why T‐bet‐deficient Th cells fail to induce colitis in ‘T‐bet‐dependent’ recipients. IFN‐γ^+^IL‐17A^+^ Th1/17 cells have been described as proinflammatory drivers of T cell transfer colitis before 3, 9. Our own previous data, however, demonstrate that neither IFN‐γ nor IL‐17A expression by the transferred Th cells is critical for colitis development, but rather the expression of chemokines recruiting pro‐inflammatory myeloid cells by the Th cells 5. How this is controlled by the microbiota is still unclear.

Recently, it has emerged that the susceptibility to inflammatory diseases is not determined by the host genome alone but by its interaction with the microbiome 24. *Helicobacter hepaticus* and *Bacteroides* for example were shown to induce intestinal inflammation only in hosts that are genetically susceptible 13, 25. Reciprocally, our results show that genetic deficiency for T‐bet in T cells alleviates colitis only in the context of a certain microbiota. Thus, host genome – microbiota interactions not only explain variability in disease susceptibility, but likely also the discrepancies in the results of genetic studies in mice – as we illustrate here for the T‐bet requirement in T cell transfer colitis.

Our findings suggest that IBD may comprise many distinct pathologies that depend on the patient's genetic predisposition and microbiota composition. In this scenario, T‐bet‐dependent Th1 cells likely represent one out of many disease‐driving T cell subpopulations resulting in a distinct IBD subgroup. In support of this, a recent study revealed that IBD comprises a continuous spectrum of diseases rather than just Crohn's disease and ulcerative colitis 26. Specifically, colonic Crohn's disease was found to be as different from ileal Crohn's disease as it is from ulcerative colitis again suggesting an important role for the microbiota – which is different between ileum and colon – in shaping these pathologies. Finally, the heterogeneous response of IBD patients to various biologicals targeting IFN‐γ, TNF, IL‐12/23, and integrin α4β7, points to diverse inflammatory mechanisms being at work 27.

## Concluding remarks

Our study explains the disparate results regarding the requirement for T‐bet for the induction of CD4+ T cell‐mediated intestinal inflammation and highlights that the composition of the intestinal microbiota can determine the molecular pathways that lead to chronic inflammation. These findings not only suggest that human IBD pathophysiology may be highly individual and microbiota‐dependent but also again stress the importance of standardizing the microbiota in animal experiments.

## Materials and methods

### Mice

C57BL6/J, *Rag1* ^−/−^, and *Tbx21* ^−/−^ mice were housed and bred under specific pathogen‐free conditions in individually ventilated cages. *Rag1* ^−/−^ mice were initially purchased from the Jackson Laboratory (USA) and then bred at Charles River (Sulzfeld, Germany). Absence (in ‘T‐bet‐dependent’ *Rag1* ^−/−^ mice) or presence (in ‘T‐bet‐independent’ *Rag1* ^−/−^ mice) of *Helicobacter species* (*H. spp*) and segmented filamentous bacteria (SFB) was confirmed at the beginning and at the end of the experiment by PCR from fecal DNA with the following primer pairs: *H. spp* Fw: 5′‐ctatgacgggtatccggc‐3′**,** Rv: 5′‐attccacctacctctccca‐3′, SFB Fw: 5′‐gacgctgaggcatgagagcat‐3′**,** Rv: 5′‐gacggcacggattgttattca‐3′. T‐bet‐independent *Rag1* ^−/−^ mice were colonized by oral gavage with a fecal bacterial suspension tested positive for *H. spp* and SFB at least 2 weeks prior to T cell transfer 5. Mice were handled in accordance with good animal practice as defined by the German animal welfare bodies. All experiments were approved by the regulatory office „Landesamt für Gesundheit und Soziales“ in Berlin, Germany under the permit number G0300/11.

### Colitis induction

Colitis was induced as published before 2. Briefly, CD4^+^ T cells from spleen and lymph nodes of WT or *Tbx21* ^−/−^ C57BL/6J donors were purified by high‐gradient magnetic cell sorting (MACS) using mouse CD4 direct beads (L3T4, Miltenyi Biotec). Viable CD4^+^CD45RB^hi^CD25^−^ cells were isolated by fluorescence‐activated cell sorting (FACS) with a FACSAria I (BD Biosciences). 4 × 10^5^ cells were injected i.v. into each *Rag1* ^−/−^ recipient. Different experimental groups (recipients of WT and *Tbx21* ^−/−^ Th cells) were cohoused in the same cages. Mice were sacrificed about 6 weeks after transfer.

### Histology

Colons were fixed in 4% paraformaldehyde at 4°C in the “Swiss roll” formation overnight. After washing with PBS and dewatering, colons were embedded in paraffin. Sections were stained with hematoxylin and eosin. Colitis histopathology was scored in a blinded fashion as published before 5.

### Isolation of lamina propria leukocytes

Lamina propria leukocytes (LPL) were isolated from colon and small intestine as described before 5. In brief, intestines were freed from fat, opened longitudinally and washed with PBS. The epithelial layer was stripped off in two rounds of incubation in calcium/magnesium‐free HBSS with 5 mM EDTA and 10 mM HEPES for 20 min at 37 °C. To obtain a single cell suspension of the mucosa, intestines were minced into small pieces and incubated 3 times for 20 min at 37 °C with 0.5 mg/ml Collagenase D (Roche), 0.5 mg/ml DNase I (Sigma Aldrich) and 0.05 U/ml Dispase (BD Biosciences). For small intestines, LPL were separated from debris by centrifugation over a Percoll gradient.

### Restimulation and flow cytometry

For intracellular cytokine staining, cells were restimulated with 10 ng/mL PMA and 1 μg/mL ionomycin in IMDM medium, containing 10% FCS at 5 × 10^6^‐1 × 10^7^ cells/mL for a total of 4 h 28. After 1 h, brefeldin A was added to a final concentration of 5 μg/mL. Cells were stained with a fixable live/dead discrimination dye for 20 min on ice, fixed with Cytofix/Cytoperm buffer (BD) and stained in 0.5% w/v Saponin for 20 min on ice. For transcription factor staining, cells were stained with the fixable live/dead discrimination dye directly *ex vivo*, fixed with the Foxp3 staining buffer kit (eBioscience) for 1 h on ice and stained in the 1× perm buffer from the Foxp3 staining buffer kit for 1 h on ice. Samples were acquired on an MACS Quant (Miltenyi Biotec). The following antibodies and reagents were used (clone, supplier): CD3ε APC‐eFluor® 780 (145‐2C11, eBioscience), CD4 Pe‐Cy7 (RM4‐5, eBioscience), CD45RB PE (16A, BD Biosciences), CD25 APC (PC61, Biolegend), IFN‐γ PerCP‐Cy5.5 (XMG1.2, eBioscience), IL‐17A FITC (TC11‐18H10.1, Biolegend), ROR‐γt PE (Q31‐378, BD Biosciences), T‐bet Alexa Fluor® 647 (4B10, Biolegend).

### 16S rDNA sequencing of fecal bacteria

DNA isolation and 16S sequencing were performed as described previously 29. Raw sequence data were deposited at the NCBI Sequence Read Archive (SRA) under the accession number SRP078391 (T‐bet‐independent recipients: SRP069847 from Ref. 5). Combined reads from both projects were classified using the ribosomal database project “Classifier” tool with a confidence cutoff of 50% 30. The copy number‐adjusted counts were agglomerated to bacterial families and genera and plotted as ratio to the total counts of the bacterial kingdom. For the calculation of Shannon index and rarefaction analysis, the RDP pipeline was used including the tools “Aligner”, “Complete Linkage Clustering”, “Shannon & Chao1 index”, and “Rarefaction” using 5000 reads per sample 31.

To estimate beta diversity, Bray‐Curtis distances were computed by the ‘vegan’ package after resampling of the samples to equal sizes 32. Principal Coordinates Analysis (PCoA) was performed by RDPutils using the Bray‐Curtis distance 33. Linear discriminant analysis effect size 10 and Random Forest (RF) 11 approaches were used to identify the taxa responsible for the differences between ‘T‐bet‐dependent’ and ‘T‐bet‐independent’ *Rag1* ^−/−^ mice before and after colitis induction 11. Copy number‐adjusted bacterial rDNA counts were agglomerated at the genus level and normalized to total bacterial counts. LEfSe was applied with default parameter settings based on bacterial frequencies scaled by 1M without further normalization. To account for the highly diverse compositions of microbiomes between ‘T‐bet‐dependent’ and ‘T‐bet‐independent’ *Rag1* ^−/−^ mice solely taxa with frequencies above 0.1% or 1% in at least one investigated sample were considered. For the significance a LDA threshold > 2 was used. The RFs were constructed based on the frequencies of classified taxa at genus level before and after colitis induction using 1M trees in each RF. To account for the highly diverse compositions three randomly chosen taxa were used in each split and solely taxa with read frequencies above 0.1 or 1% in all samples of at least one of the groups were considered. The impact was measured by the mean decrease of the Gini coefficient as well as the estimated accuracy of the prediction after perturbation of the frequencies for each considered taxon.

Differences in the abundance of metabolic pathways were determined using PICRUSt 16 and LEfSe. OTU tables for PICRUSt were prepared with QIIME 34 by assigning taxonomies based on Greengenes reference OTUs 13–5 35 to raw sequences with the RDP method. Reference OTUs were picked with a similarity of 0.97. Subsequently, OTU‐tables were down‐sampled to the smallest library size, adjusted to copy numbers and subjected to KEGG‐ortholog prediction by PICRUSt. Orthologs involved in metabolism were further normalized to 1M counts and summarized in KEGG categories. LEfSe abundance tables were constructed by KEGG level 1–3 categories and redundantly augmented by the normalized ortholog counts. LEfSe was applied with default parameter settings without further normalization.

### Data presentation and statistical evaluation

Graphs depict mean ± SEM unless stated otherwise. Statistical analysis was done with Graph pad prism version 5 using a one‐way ANOVA followed by the post‐test indicated in the figure legend. For the comparison of two groups, the Student's t‐test for independent samples or the Mann–Whitney‐U‐test for independent samples was used depending on the group sizes.

## Conflict of interest

The authors declare no commercial or financial conflict of interest.

**Abbreviations**
CD: Cluster of Differentiation
IBD: Inflammatory bowel disease
IFN‐γ: Interferon gamma
IL: Interleukin
LEfSe: Linear discriminant analysis effect size
PICRUSt: Phylogenetic investigation of communities by reconstruction of unobserved states
Rag: Recombination‐activating gene
ROR‐γt: Retinoic acid receptor‐related orphan receptor gamma t
SFB: Segmented filamentous bacteria
T‐bet: T‐box expressed in T cells
Th cell: T helper cell
TNF: Tumor necrosis factor
WT: wild type

## Supporting information

Supporting Information

Peer review correspondence
