# Supplementary material for: The intestinal microbiota determines the colitis‐inducing potential of T‐bet‐deficient Th cells in mice
Source: Eur J Immunol. 2017 Sep 29;48(1):161–7. doi: 10.1002/eji.201747100 (PMC5813160; doi:10.1002/eji.201747100)
Supplement: Supplementary file 1 — Supporting Information [file EJI-48-161-s001.pdf]

European Journal of Immunology

Supporting Information
for
DOI 10.1002/eji.201747100

Jakob Zimmermann, Pawel Durek, Anja A. Kühl, Florian Schattenberg, Patrick Maschmeyer, Francesco Siracusa, Katrin Lehmann, Kerstin Westendorf, Melanie Weber, René Riedel, Susann Müller, Andreas Radbruch and Hyun-Dong Chang

The intestinal microbiota determines the colitis-inducing potential of T-bet-deficient Th cells in mice

© 2017 The Authors. *European Journal of Immunology* published by WILEY-VCH Verlag GmbH & Co. KGaA, Weinheim.

The intestinal microbiota determines the colitis-inducing potential of T-bet-deficient Th cells

Supporting Information

Jakob Zimmermann[1,4], Pawel Durek[1], Anja A. Kühl[2], Florian Schattenberg[3], Patrick Maschmeyer[1], Francesco Siracusa[1], Katrin Lehmann[1], Kerstin Westendorf[1], Melanie Weber[1], René Riedel[1,5], Susann Müller[3], Andreas Radbruch[1]*, Hyun-Dong Chang[1]*°

1) Deutsches Rheumaforschungszentrum Berlin (DRFZ), an Institute of the Leibniz Association, Charitéplatz 1, 10117 Berlin, Germany
2) Charité Universitätsmedizin, Campus Benjamin Franklin, Hindenburgdamm 30, 12203 Berlin, Germany
3) Helmholtzzentrum für Umweltforschung (UFZ) Leipzig, Department Umweltmikrobiologie, Permoserstrasse 15, 04318 Leipzig, Germany
4) Maurice Müller Laboratories (DKF), Universitätsklinik für Viszerale Chirurgie & Medizin Inselspital, University of Bern, Murtenstrasse 35, 3008 Bern, Switzerland.
5) Max Planck Institute for Evolutionary Biology, August-Thienemann-Straße 2, 24302 Plön, Germany

*Authors share co-senior authorship.

°Corresponding author:

Dr. Hyun-Dong Chang

phone: 0049/(0)30/28460-683, fax: 0049/(0)30/28460-603, e-mail: chang@drfz.de

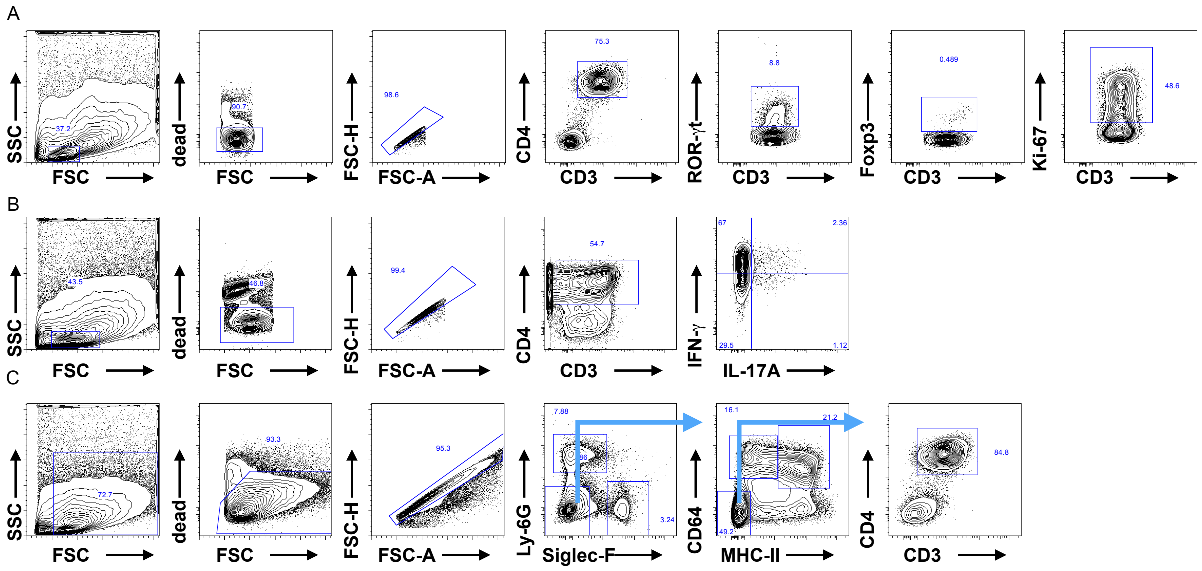

Supporting Information Figure 1 – Flow cytometry gating strategies

Naïve CD4^{+}CD45RB^{hi}CD25^{-} WT or *Tbx21*$^{-/-}$ CD4 T cells were transferred into *Rag1*$^{-/-}$ recipients. Upon analysis, (A) transcription factor expression was measured on colonic lymphocyte-shaped (FSC/SSC), viable, single, CD3+CD4+ Th cells. (B) Following PMA/ionomycin-mediated restimulation, cytokine expression was quantified on colonic lymphocyte-shaped (FSC/SSC), viable, single, CD3+CD4+ Th cells. (C) The colonic inflammatory infiltrate was characterized by enumerating leukocyte-shaped (FSC/SSC), viable, single Ly-6G+ neutrophils, Siglec-F+ eosinophils, (Ly-6G-Siglec-F-) CD64+MHC-II+ Macrophages, CD64+MHC-II- monocytes, and (Ly-6G-Siglec-F-CD64-MHC-II-) CD3+CD4+ Th cells.

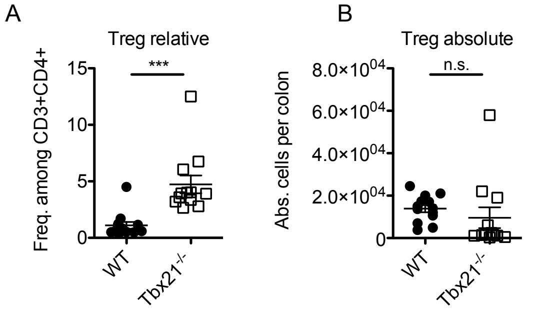

Supporting Information Figure 2 – Abundance of Foxp3+ regulatory T cells in colitis

Naïve $CD4^{+}CD45RB^{hi}CD25^{-}$ WT or *Tbx21*$^{-/-}$ CD4 T cells were transferred into *Rag1*$^{-/-}$ recipients directly from Charles River ('T-bet-dependent' *Rag1*$^{-/-}$). Abundance of colonic Foxp3+ Treg in colitis (day 40-44) is indicated as the frequency among CD3+CD4+ T helper cells (A) and as absolute numbers per colon (B). n=12-13 per group from 3 independent experiments, *** = $p < 0.001$ by Student's t-test for independent samples.

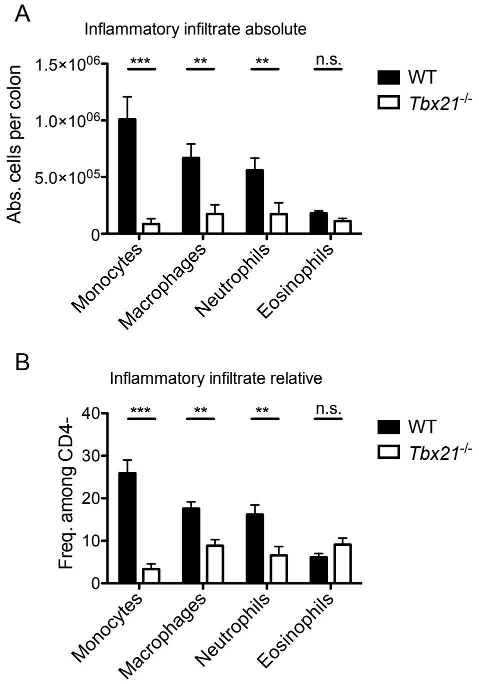

Supporting Information Figure 3 – Colonic inflammatory infiltrate in 'T-bet-dependent' *Rag1*$^{-/-}$ recipients Naïve CD4^{+}CD45RB^{hi}CD25^{-} WT or *Tbx21*$^{-/-}$ CD4 T cells were transferred into *Rag1*$^{-/-}$ recipients directly from Charles River ('T-bet-dependent' *Rag1*$^{-/-}$). On day 40-44 colonic monocytes (CD64+Ly-6C^{hi}), macrophages (CD64+Ly-6C^{low}), neutrophils (Ly-6G+), and eosinophils (Siglec-F+) were analyzed by flow cytometry and plotted as absolute number of cells per colon (A) or relative frequency among CD4- cells (B). n=12-13 mice per group from 3 independent experiments. ** = $p<0.01$ and *** = $p<0.001$ by Student's t test for independent samples.

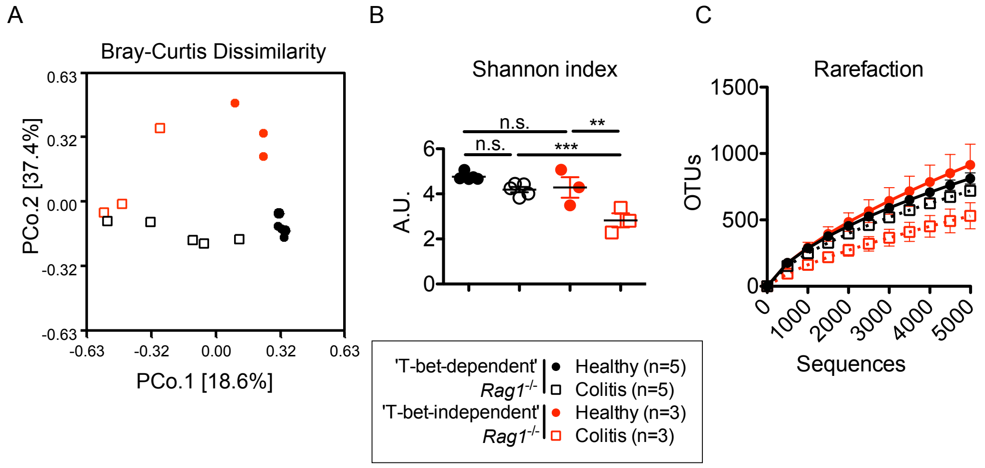

Supporting Information Figure 4 Alpha and beta diversity of 'T-bet-dependent' and 'T-bet-independent' *Rag1*$^{-/-}$ recipient mice Naïve $CD4^{+}CD45RB^{hi}CD25^{-}$ WT or *Tbx21*$^{-/-}$ CD4 T cells were transferred into *Rag1*$^{-/-}$ recipients directly from Charles River ('T-bet-dependent' *Rag1*$^{-/-}$) or after colonization with fecal microbiota from the DRFZ animal colony ('T-bet-independent' *Rag1*$^{-/-}$). Fecal microbiomes were determined by 16S rDNA sequencing for n=5 'T-bet-dependent' *Rag1*$^{-/-}$ mice from one experiment and n=3 'T-bet-independent' *Rag1*$^{-/-}$ mice from Supporting Information reference [1] before ('Healthy') and after ('Colitis') transfer of WT Th cells ('T-bet-dependent' *Rag1*$^{-/-}$: day 40, 'T-bet-independent' *Rag1*$^{-/-}$: day 12-15). (A) Bray-Curtis dissimilarity, (B) Shannon index and (C) Rarefaction were calculated with the Ribosomal Database Project (RDP) pipeline at a clustering threshold of 97% [2]. **p<0.01, ***p<0.001 by One-Way ANOVA and Newman-Keuls post test.

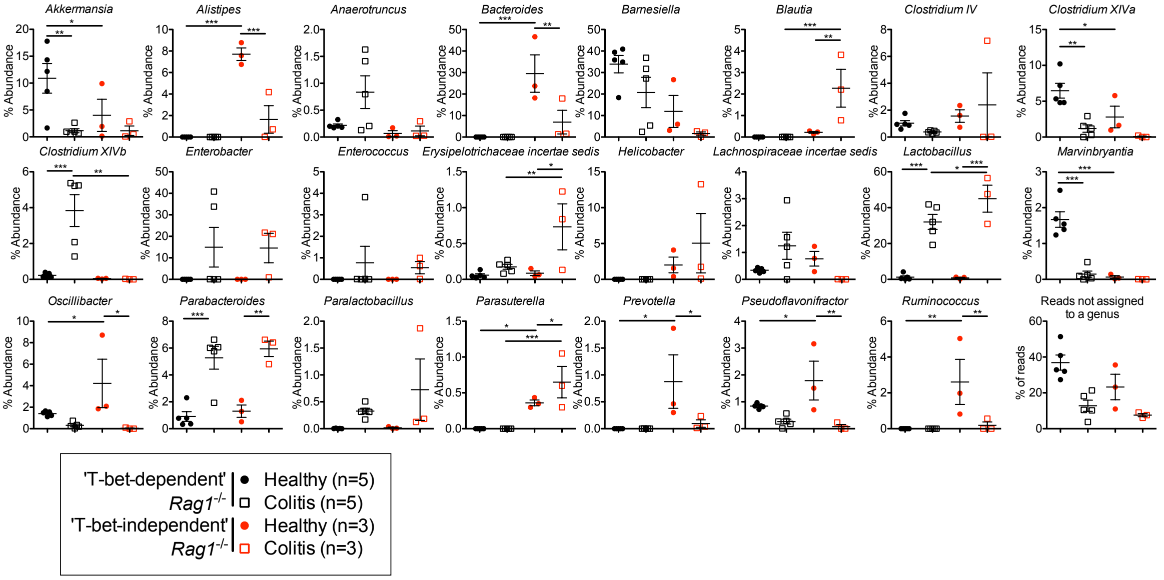

Supporting Information Figure 5 Genus level microbiome analysis from 'T-bet-dependent' and 'T-bet-independent' *Rag1*$^{-/-}$ recipient mice

Naïve CD4^{+}CD45RB^{hi}CD25^{-} WT or *Tbx21*$^{-/-}$ CD4 T cells were transferred into *Rag1*$^{-/-}$ recipients directly from Charles River ('T-bet-dependent' *Rag1*$^{-/-}$) or after colonization with fecal microbiota from the DRFZ animal colony ('T-bet-independent' *Rag1*$^{-/-}$). Fecal microbiomes were determined by 16S rDNA sequencing for n=5 'T-bet-dependent' *Rag1*$^{-/-}$ mice from one experiment and n=3 'T-bet-independent' *Rag1*$^{-/-}$ mice from Supporting Information reference [1] before ('Healthy') and after ('Colitis') transfer of WT Th cells ('T-bet-dependent' *Rag1*$^{-/-}$: day 40, 'T-bet-independent' *Rag1*$^{-/-}$: day 12-15). Genera with a threshold read frequency of at least 1% in at least one of the samples were analyzed by one-way-ANOVA followed by Newman-Keuls post test with *p<0.05, **p<0.01, and ***p<0.001. $\bar{x}$±SEM are indicated.

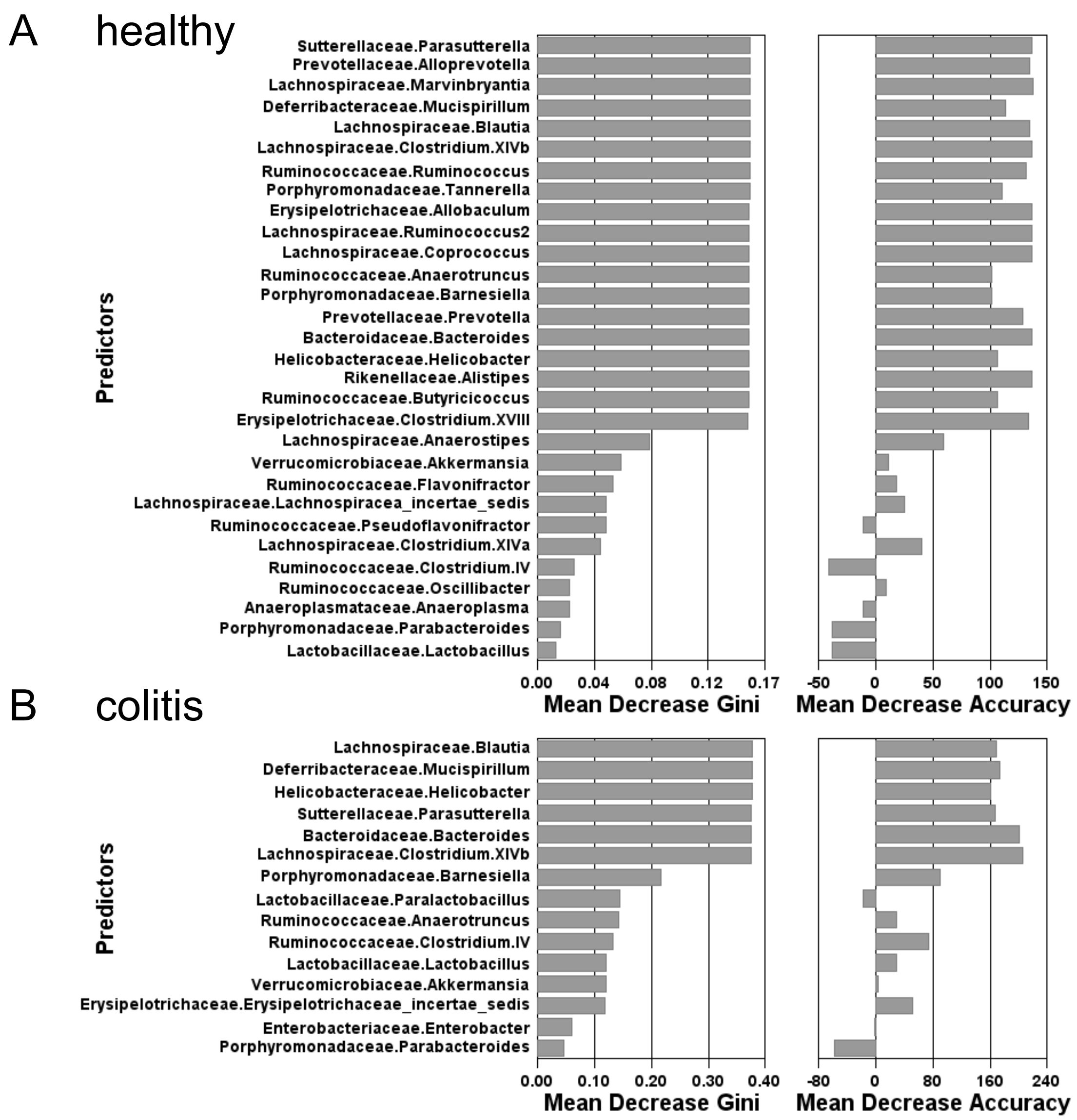

Supporting Information Figure 6 – Random forest analysis of 'T-bet-dependent' and 'T-bet-independent' *Rag1*$^{-/-}$ microbiomes

Healthy (A) and colitic (B) 'T-bet-dependent' and 'T-bet-independent' *Rag1*$^{-/-}$ microbiomes from Figure 2 were subjected to random forest analysis to identify distinguishing taxa. Taxa were filtered for those with a read frequency of 0.1% or higher in all samples of at least one group. The mean decrease in the Gini coefficient (measure for the average gain of purity by splits of a given taxa) and the mean decrease in the prediction accuracy (estimation of how well an unknown sample would be assigned to the correct group) caused by permutation of the respective frequencies of the given taxa among the samples are indicated. Higher values mean higher impact on the prediction.

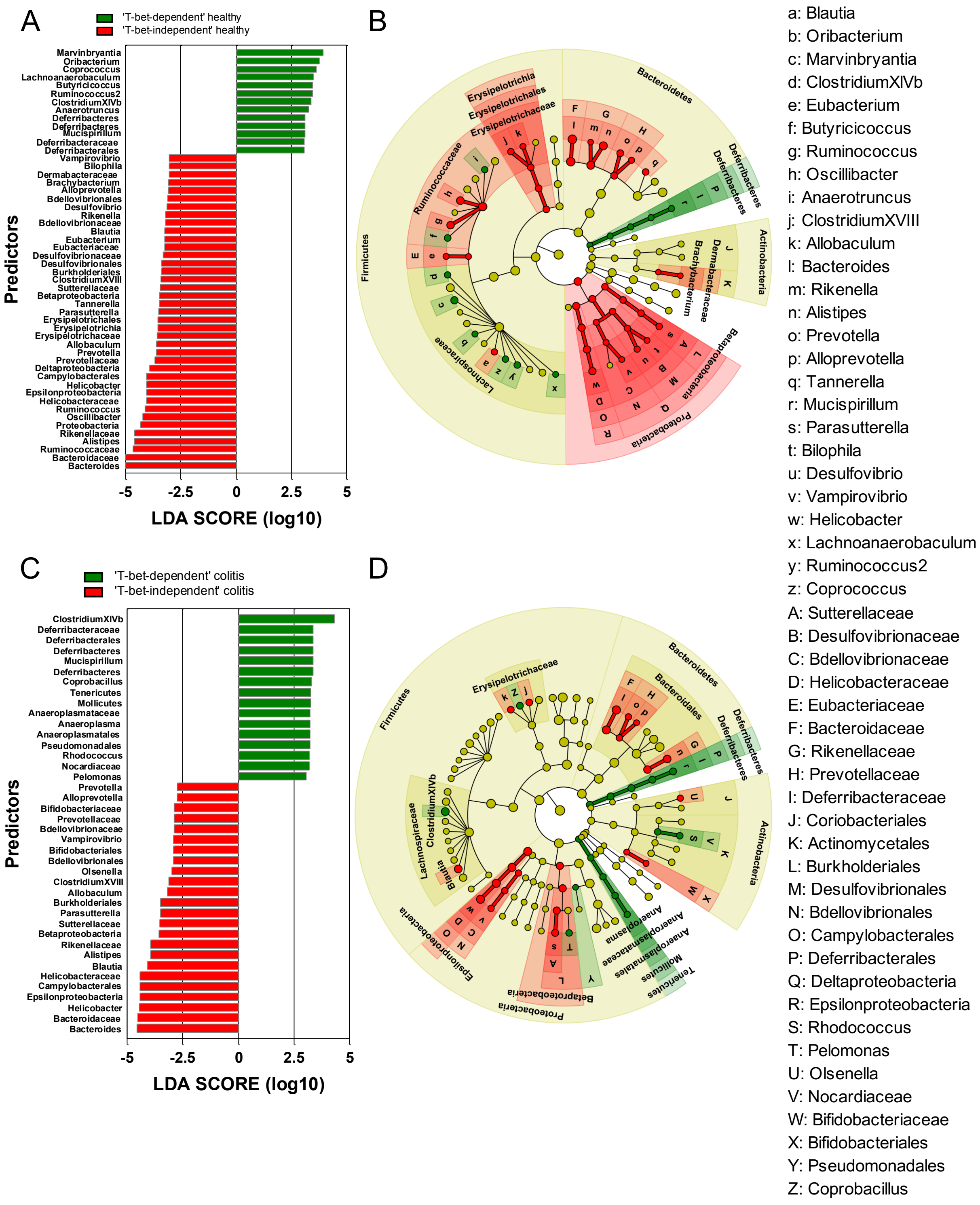

Supporting Information Figure 7 LEfSe microbiome analysis with 0.1% abundance threshold

Fecal microbiomes of mice from Figure 2 were determined by 16S rDNA sequencing for n=5 'T-bet-dependent' *Rag1*$^{-/-}$ mice and n=3 'T-bet-independent' *Rag1*$^{-/-}$ mice from Supporting Information reference [3] before ('Healthy') and after ('Colitis') transfer of WT Th cells ('T-

bet-dependent' *Rag1*$^{-/-}$: day 40, 'T-bet-independent' *Rag1*$^{-/-}$: day 12-15). (A and C) Linear discriminant analysis (LDA) scores of taxa significantly enriched in 'T-bet-dependent' (green) or 'T-bet-independent' (red) microbiomes (A - healthy, C - colitis). Taxa with a relative abundance of at least 0.1% in at least one sample were included. LDA scores ≥ 2 were considered significant. (B and D) Cladograms showing the phylogenetic relationship among the analyzed taxa with dot size representing average abundance of the taxa.

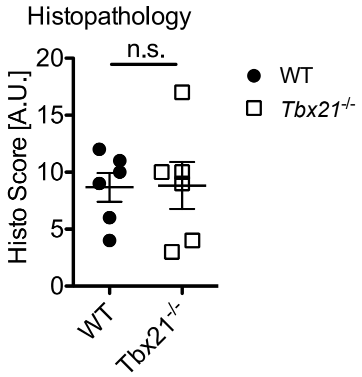

Supporting Information Figure 8 WT and T-bet-deficient Th cells induce colitis of similar severity in *Rag1*$^{-/-}$ recipients negative for *Helicobacter species*

Naïve CD4^{+}CD45RB^{hi}CD25^{-} WT or *Tbx21*$^{-/-}$ CD4 T cells were transferred into *Rag1*$^{-/-}$ with a fecal microbiota tested negative for *Helicobacter species* by PCR. Colon histopathology ($\bar{x}$±SEM) on day 38-40 is depicted for n=6 mice per group pooled from two independent experiments (n.s. = $p > 0.05$ by Mann-Whitney-U test for independent samples).

Supporting Information References

1. **Zimmermann J**, **Kuhl AA**, **Weber M**, **Grun JR**, **Loffler J**, **Haftmann C**, **Riedel R**, *et al.* T-bet expression by Th cells promotes type 1 inflammation but is dispensable for colitis. *Muc Immunol*. 2016; **9**:1487–1499.DOI: 10.1038/mi.2016.5.

2. **Kunin V**, **Engelbrektson A**, **Ochman H**, **Hugenholtz P**. Wrinkles in the rare biosphere: pyrosequencing errors can lead to artificial inflation of diversity estimates. *Environmental Microbiology*. 2010; **12**:118–123.DOI: 10.1111/j.1462-2920.2009.02051.x.

3. **Zimmermann J**, **Hübschmann T**, **Schattenberg F**, **Schumann J**, **Durek P**, **Riedel R**, **Friedrich M**, *et al.* High-resolution microbiota flow cytometry reveals dynamic colitis-associated changes in fecal bacterial composition. *Eur J Immunol*. 2016; **46**:1300–1303.DOI: 10.1002/eji.201646297.
