# Supplementary material for: The intestinal microbiota determines the colitis‐inducing potential of T‐bet‐deficient Th cells in mice
Source: Eur J Immunol. 2017 Sep 29;48(1):161–7. doi: 10.1002/eji.201747100 (PMC5813160; doi:10.1002/eji.201747100)
Supplement: Supplementary file 2 — Peer review correspondence [file EJI-48-161-s002.pdf]

European Journal of Immunology

The intestinal microbiota determines the colitis-inducing potential of T-bet-deficient Th cells in mice

Jakob Zimmermann, Pawel Durek, Anja A. Kühl, Florian Schattenberg, Patrick Maschmeyer, Francesco Siracusa, Katrin Lehmann, Kerstin Westendorf, Melanie Weber, René Riedel, Susann Müller, Andreas Radbruch and Hyun-Dong Chang

Correspondence: Dr. Hyun-Dong Chang, Deutsches Rheumaforschungszentrum Berlin (DRFZ), an Institute of the Leibniz Association, Charitéplatz 1, 10117 Berlin, Germany

---

Review Timeline:	Submission date:	24-Apr-2017
	First Editorial decision:	12-Jun-2017
	Revision received:	28-Jul-2017
	Accepted:	31-Aug-2017

Handling Executive Committee member: Prof. Iain McInnes

Please note that the correspondence below does not include the standard editorial instructions regarding preparation and submission of revised manuscripts, only the scientific revisions requested and addressed.

First Editorial Decision 12-Jun-2017

Dear Dr. Chang,

We apologize for the delay in the peer review process of your recently submitted manuscript ID eji.201747100 entitled "The intestinal microbiota determines the colitis-inducing potential of T-bet-deficient Th cells" which you submitted to the European Journal of Immunology. Please find the comments of the referees included at the bottom of this letter.

A revised version of your manuscript that takes into account the comments of the referees will be reconsidered for publication. Our Executive Editor strongly encourages you to use LEfSe plots in your

analysis as suggested by Referee 2. Should you disagree with any of the referees concerns, you should address this in your point-by-point response and provide solid scientific reasons for why you will not make the requested changes.

You should also pay close attention to the editorial comments included below. **In particular, please edit your figure legends to follow Journal standards as outlined in the editorial comments. Failure to do this will result in delays in the re-review process.**

Please note that submitting a revision of your manuscript does not guarantee eventual acceptance, and that your revision will be re-reviewed by the referees before a decision is rendered.

If the revision of the paper is expected to take more than three months, please inform the editorial office. Revisions taking longer than six months may be assessed by new referees to ensure the relevance and timeliness of the data.

Once again, thank you for submitting your manuscript to European Journal of Immunology and we look forward to receiving your revision.

Yours sincerely,
Eloho Etemire

On behalf of
Prof. Francesco Annunziato

Dr. Eloho Etemire
Editorial Office
European Journal of Immunology
e-mail: ejied@wiley.com
www.eji-journal.eu

****

European Journal of Immunology

Reviewer: 1

Comments to the Author

The authors investigate the widely used T cell transfer colitis model, originally established by Powrie and colleagues. They show that depending on their microbiota composition recipient animals are differentially susceptible to colitis induced by T bet-proficient and -deficient naive T cells. These data emphasize the importance of host-microbiome interactions in colitis induction and development and clarify an outstanding issue with respect to the Powrie model, i.e conflicting data on the requirement of Th1 T cells.

The manuscript is very concisely written and all conclusions are supported by clearly presented data. I recommend publication of this study.

minor comments and suggestions for edits and additions

• Figure 1E-G shows that Tbet-deficient T cells express higher levels of rorgt and il17 upon transfer, as also shown in the previous publication by Zimmermann J et al (ref 3).

a. Are the Rorgt+ cells only IL17A+ or are they IFNy+ as well? Are some of them double positive for IFNg and IL17A? – This is easy to solve by gating – gate on the Rorgt+ cells and check their cytokine staining

b. Are the rorgt+ cells also foxp3+? There have been many reports of rorgt foxp3 double positive Tregs. This could be addressed by staining the cells for foxp3. In the previous publication there was a staining for foxp3 but not a double staining.

• The authors show a different susceptibility to colitis as a result of different microbiome composition. In their previous publication they emphasized the importance of the myeloid lineage to the colitis phenotype, and specifically the interaction between myeloid cells and transferred T cells. Can they comment on if there any effect of the microbiota on the myeloid cell composition or function in these mice, prior or following colitis induction?

European Journal of Immunology

Reviewer: 2

Comments to the Author

This study shows that the gut microbiome drives colitis-inducing potential of T-bet deficient Th cells in RAg-/- mice. Since there has been conflicting data on the role of Tbet+ T cells in RAG-/-, this study clarifies that the microbial composition of mice are different at various facilities and show through fecal transplantation that its the type of microbes present that drive this phenotype through the Tbet cells. The authors suggest that its the type of microbes present evident based on fecal transplant however, it can not be rules out that its metabolites or other functional microbial derived products. It would be useful to measure short chain fatty acids. As well, they suggest the mechanism is through Th17 cells yet segmented filamentous bacteria drive Th17 expansion in the gut but these microbes didn't appear in the microbiome analysis. Although the authors do state that mice were SFB negative and then tested positive post-transplant. I find this confusing so it needs better explanation. This manuscript would be strengthen by:

1) use Lefse plots to show differences in bacterial taxa
2) at least use predictive software like PICRUST to look at bacterial function
3) measure short chain fatty acids in the microbiomes
4) the sample size for the microbiome analysis is 3-5 which is unacceptable. The sample size must be >5 and cage effects must be accounted for in the design (is they why you co-housed?)
5) clarify the role of SFB

<u>First Revision – authors' response 28-Jul-2017</u>

Dear Prof Annunziato,

thank you for the critical assessment of our manuscript „The intestinal microbiota determines the colitis-inducing potential of T-bet-deficient Th cells".

We are delighted that both reviewers share our enthusiasm about the study.

We have carefully taken the reviewer's advice into account and edited the manuscript accordingly. Please find below our point-by-point response to the reviewer's comments.

European Journal of Immunology

Reviewer 1

Comments to the Author

The authors investigate the widely used T cell transfer colitis model, originally established by Powrie and colleagues. They show that depending on their microbiota composition recipient animals are differentially susceptible to colitis induced by T bet-proficient and -deficient naive T cells. These data emphasize the importance of host-microbiome interactions in colitis induction and development and clarify an outstanding issue with respect to the Powrie model, i.e conflicting data on the requirement of Th1 T cells. The manuscript is very concisely written and all conclusions are supported by clearly presented data. I recommend publication of this study. minor comments and suggestions for edits and additions

Comment 1

Figure 1E-G shows that Tbet-deficient T cells express higher levels of rorgt and il17 upon transfer, as also shown in the previous publication by Zimmermann J et al (ref 3). a. Are the Rorgt+ cells only IL17A+ or are they IFNy+ as well? Are some of them double positive for IFNg and IL17A? – This is easy to solve by gating – gate on the Rorgt+ cells and check their cytokine staining

<u>Author's response</u>

For technical reasons, we have measured ROR-γt and cytokine expression of the T cells in separate flow cytometry panels and are thus unfortunately unable to gate on ROR-γt+ cells to directly comment on their cytokine profile. However, the question of the reviewer can be answered indirectly from the cytokine data presented in Figure 1 H: In both T-bet-dependent and T-bet-independent recipients, T-bet-deficient Th cells expressed IL-17A at increased frequencies compared with WT. However, whereas in T-bet-independent recipients half of these IL-17A+ T cells coexpressed IFN-γ, only 25% of IL-17A+ T cells coexpressed IFN-γ in T-bet-dependent recipients.

Comment 2

Are the rorgt+ cells also foxp3+? There have been many reports of rorgt foxp3 double positive Tregs. This could be addressed by staining the cells for foxp3. In the previous publication there was a staining for foxp3 but not a double staining.

Author's response

The frequency of Foxp3+ Treg is generally rather low (~1%) upon transfer of naïve CD45RB^{hi}CD25^{-} T helper cells. It is, however, significantly increased after transfer of T-bet-deficient Th cells specifically in T-bet-dependent *Rag1*$^{-/-}$ recipients. Nevertheless, the absolute number of Foxp3+ Treg in these mice is similar for WT and T-bet-deficient Th cells, suggesting that it is not an increased Treg compartment that prevents intestinal inflammation in T-bet-dependent *Rag*$^{-/-}$ recipients following transfer of T-bet-deficient Th cells. We have included this information now in Supporting Information Fig. 2

Moreover, most of the ROR-γt+ cells were Foxp3-. In the T-bet-dependent *Rag1*$^{-/-}$ recipients analyzed here, only 0.8% of WT ROR-γt+ T cells and 3.4% of T-bet-deficient ROR-γt+ T cells were also Foxp3 positive. Thus, the overwhelming majority of ROR-γt+ T cells were bona fide Th17 cells.

Comment 3

The authors show a different susceptibility to colitis as a result of different microbiome composition. In their previous publication they emphasized the importance of the myeloid lineage to the colitis phenotype, and specifically the interaction between myeloid cells and transferred T cells. Can they comment on if there any effect of the microbiota on the myeloid cell composition or function in these mice, prior or following colitis induction?

Author's response

We have included the information about the composition of the myeloid compartment in the revised version of the manuscript as Supporting Information Fig. 3. In 'T-bet-dependent' *Rag1*$^{-/-}$ recipients, monocytes, macrophages, and neutrophils were strongly reduced in colitis induced by T-bet-deficient compared with WT Th cells. This contrasts our findings with T-bet-independent *Rag1*$^{-/-}$ recipients, where T-bet-deficient Th cells recruited similar amounts of neutrophils and even increased numbers of eosinophils to the colon when compared with WT Th cells. Myeloid effector cells are recruited to the colon by the colitogenic T cells (Griseri et al., Immunity, 2015; Zimmermann et al., Mucosal Immunology, 2016) and we thus think that the reduced numbers of myeloid cells in colitis induced by T-bet-deficient

European Journal of Immunology

Th cells in T-bet-dependent recipients is a secondary effect of reduced numbers of T cells rather than directly mediated by the microbiota.

Reviewer 2

Comments to the Author

This study shows that the gut microbiome drives colitis-inducing potential of T-bet deficient Th cells in Rag-/- mice. Since there has been conflicting data on the role of Tbet+ T cells in Rag-/-, this study clarifies that the microbial composition of mice are different at various facilities and show through fecal transplantation that its the type of microbes present that drive this phenotype through the Tbet cells.

Comment 1

The authors suggest that its the type of microbes present evident based on fecal transplant however, it cannot be ruled out that it is metabolites or other functional microbial derived products.

Author's response

We agree with the reviewer that the differential abundance of microbiota-derived metabolites could shape the susceptibility to colitis induced by T-bet-deficient Th cells. However, differential metabolite abundance would be a secondary consequence of the altered composition and thus the primary causal reason for differential colitis susceptibility would still be microbiota composition. We have clarified that the exact mechanism of how the microbiota influences colitis susceptibility remains to be determined on page 6 lines 5-8: "Overall, it remains to be determined which bacterial taxa differentially activate T-bet-sufficient or T-bet-deficient Th cells to induce colitis and how they do it, e.g. by microbiota-derived metabolites or direct interaction with the host."

Comment 2

It would be useful to measure short chain fatty acids. As well, they suggest the mechanism is through Th17 cells yet segmented filamentous bacteria drive Th17 expansion in the gut but these microbes didn't appear in the microbiome analysis. Although the authors do state that mice were SFB negative and then

tested positive post-transplant. I find this confusing so it needs better explanation. This manuscript would be strengthen by:

1) use Lefse plots to show differences in bacterial taxa

Author’s response

We thank the reviewer for this helpful suggestion and have incorporated LefSE plots in Fig. 2 and Supporting Information Fig. 7 of the revised version of the manuscript.

2) at least use predictive software like PICRUST to look at bacterial function

Author’s response

We have used PICRUSt to predict the differential abundance of pathways in the two metagenomes, susceptible and resistant to colitis induced by T-bet-deficient Th cells. ‘T-bet-independent’ mice revealed many pathways that were absent from ‘T-bet-dependent’ microbiomes thus reflecting the increased number of different bacterial families in these mice. We have added the PICRUSt analysis in Fig. 2E. However, we would like to stress, that it remains highly speculative whether and which differentially abundant pathways are causative for the difference in colitis susceptibility.

3) measure short chain fatty acids in the microbiomes

Author’s response

Short chain fatty acids produced mainly by *Clostridia* (Atarashi et al., Science, 2011) were shown to regulate the numbers of colonic Tregs and suppress colitis (Smith et al., Science, 2013). However, as we now demonstrate in Supporting Information Fig. 2, the number of colonic regulatory T cells was similar in ‘T-bet-dependent’ and ‘T-bet-independent’ *Rag1*$^{-/-}$ recipients. Moreover, Smith and colleagues found that SCFA reduced colitis severity when regulatory T cells were cotransferred along with the naïve

colitogenic T cells. However, when only naïve T cells were transferred – as we have done in our manuscript – there was no effect of SCFA on colitis severity (Smith et al., Science, 2013 Figure S20B). In conclusion, we think that the measurement of SCFA in the intestinal content or feces would not substantiate the conclusions of the manuscript.

Comment 3

The sample size for the microbiome analysis is 3-5 which is unacceptable. The sample size must be >5 and cage effects must be accounted for in the design (is they why you co-housed?)

Author's response

Despite the relatively small sample size, our microbiome analyses between 'T-bet-dependent' and 'T-bet-independent' microbiomes, revealed statistically significant differences. We can thus conclude that the analysis is sufficiently powered to identify the signature taxa that distinguish the two microbiomes. Cage effects are accounted for in both the colitis severity experiments and the microbiome analyses: In the former, *Rag1*$^{-/-}$ mice receiving either WT or T-bet-deficient Th cells were always cohoused in the same cage. Obviously, however, 'T-bet-dependent' and 'T-bet-independent' *Rag1*$^{-/-}$ recipients were housed in different cages to prevent exchange of their microbiota. For the microbiome analysis, feces from two different cages ('T-bet-independent') and three different cages ('T-bet-dependent') were used.

Comment 4

Clarify the role of SFB

Author's response

We have included the information about the colonization status of the mice with SFB as determined by conventional PCR from fecal DNA on page 5, lines 15-22. SFB are absent from the remainder of the microbiome analysis as our 16S sequencing pipeline is unable to resolve SFB on the genus level. We do not think that colitis induction by T-bet-deficient Th cells selectively in 'T-bet-independent' *Rag1*$^{-/-}$ mice is due to Th17 cells: Our own previous results demonstrated that IL-17 is not pathogenic but rather protective in colitis induced by WT or T-bet-deficient Th cells (Zimmermann et al., Muc Immunol, 2016).

European Journal of Immunology

In the present manuscript, we speculate that multifunctional IFN-γ+IL-17+ double-positive T cells – previously implicated in colitis pathogenesis (Harbour et al., PNAS, 2015 and Ahern et al., Immunity, 2014) – could play a role.

Second Editorial Decision 22-Aug-2017

Dear Dr. Chang,

It is a pleasure to provisionally accept your manuscript entitled "The intestinal microbiota determines the colitis-inducing potential of T-bet-deficient Th cells" for publication in the European Journal of Immunology. For final acceptance, please follow the instructions below and return the requested items as soon as possible as we cannot process your manuscript further until all items listed below are dealt with.

Please note that EJI articles are now published online a few days after final acceptance (see Accepted Articles: http://onlinelibrary.wiley.com/journal/10.1002/(ISSN)1521-4141/accepted). The files used for the Accepted Articles are the final files and information supplied by you in Manuscript Central. You should therefore check that all the information (including author names) is correct as changes will NOT be permitted until the proofs stage.

We look forward to hearing from you and thank you for submitting your manuscript to the European Journal of Immunology.

Yours sincerely,
Eloho Etemire

on behalf of
Prof. Iain McInnes

Dr. Eloho Etemire
Editorial Office
European Journal of Immunology
e-mail: ejied@wiley.com
www.eji-journal.eu
